# Supplementary material for: The Systems Biology Research Tool: evolvable open-source software
Source: BMC Syst Biol. 2008 Jun 29;2:55. doi: 10.1186/1752-0509-2-55 (PMC2446383; doi:10.1186/1752-0509-2-55)
Supplement: Additional file 1 — SBRT Archive. An archive of the current version of the Systems Biology Research Tool. [file 1752-0509-2-55-S1.zip › sbrt-1.4.0/doc/developers_guide/api/sbrt/kernel/fba/comp/FastMsFluxRangeComputer.html]

FastMsFluxRangeComputer


|  |  |  |
| --- | --- | --- |
| **Overview** \| **Package** \| **Class** \| **Use** \| **Tree** \| **Deprecated** \| **Index** \| **Help** | |  |
| **PREV CLASS**   **NEXT CLASS** | **FRAMES**    **NO FRAMES**     **All Classes** |  |
| SUMMARY: NESTED \| FIELD \| CONSTR \| METHOD | DETAIL: FIELD \| CONSTR \| METHOD |  |


---


## sbrt.kernel.fba.comp Class FastMsFluxRangeComputer

```
java.lang.Object
  sbrt.kernel.fba.comp.AbstractCvComputer
      sbrt.kernel.fba.comp.AbstractFluxIntervalComputer
          sbrt.kernel.fba.comp.FastMsFluxRangeComputer
```

**All Implemented Interfaces:**: FluxCvComputer, FluxIntervalComputer

---

``` public class FastMsFluxRangeComputer extends AbstractFluxIntervalComputer ```

This class is used to compute Mahadevan-Schilling flux intervals in a
(sometimes) faster way than `MsFluxIntervalComputer` can.

**Author:**
:   This class was written and documented by Jeremiah Wright
    while in the Wagner lab.

---

**Field Summary**

**Fields inherited from class sbrt.kernel.fba.comp.AbstractFluxIntervalComputer**

`ZERO_CUTOFF`


**Constructor Summary**

`FastMsFluxRangeComputer(FbaOptComputer computer,
java.util.Map<java.lang.String,java.util.Set<LinearComb<java.lang.String>>> fluxCaps,
LinearSystemSolution<java.lang.String> systemSolution,
double zeroCutoff)`
  
          Constructs a new flux interval computer.


| **Method Summary** | |
| --- | --- |
| `double` | `getConstraintTolerance()`             Returns the constraint tolerance used by this flux interval computer. |
| `Interval` | `getFluxConstraint(java.lang.String rxnName)`             Returns the current flux constraint on the reaction with the provided name. |
| `java.util.Map<java.lang.String,Interval>` | `getFluxConstraints()`             Returns all current flux constraints. |
| `java.util.Map<java.lang.String,Interval>` | `getIntervals()`             Computes and returns the intervals of every flux in the fluxome used to construct this flux interval computer. |
| `java.util.Map<java.lang.String,Interval>` | `getIntervals(java.util.Collection<java.lang.String> reactionNames)`             Computes and returns the flux intervals of the reactions with the provided names. |
| `ProgressMonitor` | `getProgress()`             Returns a progress monitor for this flux interval computer. |
| `Interval` | `getRxnExprConstraint(MathExpr<java.lang.String> rxnNameExp)`             Returns the current constraint on the provided expression. |
| `java.util.Map<MathExpr<java.lang.String>,Interval>` | `getRxnExprConstraints()`             Returns all current flux expression constraints. |
| `void` | `restoreFluxConstraint(java.lang.String reactionName)`             Restores the flux constraint for the reaction with the provided name to the value contained in the fluxome. |
| `void` | `restoreFluxConstraints()`             Restores the flux constraints for all reactions in the fluxome to those values contained in the fluxome. |
| `void` | `restoreFluxConstraints(java.util.Collection<java.lang.String> reactionNames)`             Restores the flux constraints for the provided reaction names to the values contained in the fluxome. |
| `void` | `restoreRxnExprConstraint(MathExpr<java.lang.String> rxnNameExpr)`             Restores the constraint on the provided flux expression to the value contained in the fluxome. |
| `void` | `restoreRxnExprConstraints()`             Restores the constraints on all flux expressions to the values contained in the fluxome. |
| `void` | `restoreRxnExprConstraints(java.util.Collection<? extends MathExpr<java.lang.String>> rxnNameExprs)`             Restores the constraints on the provided expressions to the values contained in the fluxome. |
| `void` | `setFluxConstraint(java.lang.String reactionName, Interval c)`             Changes the flux constraint of the reaction with the provided name. |
| `void` | `setFluxConstraints(java.util.Map<java.lang.String,Interval> fluxConstraints)`             Changes the flux constraints on more than one reaction. |
| `void` | `setRxnExprConstraint(MathExpr<java.lang.String> e, Interval c)`             Changes the constraint on the provided mathematical expression of fluxes. |
| `void` | `setRxnExprConstraints(java.util.Map<? extends MathExpr<java.lang.String>,Interval> constraints)`             Changes the constraints on more than one mathematical expression of fluxes. |

**Methods inherited from class sbrt.kernel.fba.comp.AbstractFluxIntervalComputer**

`getBoundedMax, getBoundedMax, getBoundedMaxWithReverseConstrained, getBoundedMin, getBoundedMin, getInterval, getIntervals, getMaximum, getMaximum, getMaxWithReverseConstrained, getMinimum, getMinimum, getReverseRxnNames, getZeroCutoff`

**Methods inherited from class sbrt.kernel.fba.comp.AbstractCvComputer**

`checkFluxConstraint, checkNumber, checkRxnExpr, checkRxnExprConstraint, checkRxnName, checkRxnNames, getFluxome, getRxnExpr, getRxnExpr`

**Methods inherited from class java.lang.Object**

`clone, equals, finalize, getClass, hashCode, notify, notifyAll, toString, wait, wait, wait`

**Methods inherited from interface sbrt.kernel.fba.comp.FluxCvComputer**

`getFluxome`

**Constructor Detail**

### FastMsFluxRangeComputer

```
public FastMsFluxRangeComputer(FbaOptComputer computer,
                               java.util.Map<java.lang.String,java.util.Set<LinearComb<java.lang.String>>> fluxCaps,
                               LinearSystemSolution<java.lang.String> systemSolution,
                               double zeroCutoff)
```

:   Constructs a new flux interval computer.

    **Parameters:**: `computer` - the computer with which all optimizations will be performed.: `fluxCaps` - the flux caps used to constrain all unbounded fluxes.: `systemSolution` - the solution to the system of linear equations formed by the fluxome contained in the provided FBA optimization computer.: `zeroCutoff` - the zero cutoff to be used by this computer.


**Method Detail**

### getConstraintTolerance

```
public double getConstraintTolerance()
```

:   Returns the constraint tolerance used by this flux interval computer.

    :   **Specified by:**: `getConstraintTolerance` in interface `FluxIntervalComputer` **Overrides:**: `getConstraintTolerance` in class `AbstractFluxIntervalComputer`
    :   **Returns:**: the constraint tolerance used by this flux interval computer.

---


### getProgress

```
public ProgressMonitor getProgress()
```

:   Returns a progress monitor for this flux interval computer.

    :   **Returns:**: a progress monitor for this flux interval computer.

---


### getIntervals

```
public java.util.Map<java.lang.String,Interval> getIntervals()
```

:   Computes and returns the intervals of every flux in the fluxome
    used to construct this flux interval computer.

    :   **Returns:**: reaction names mapped to the computed interval of their corresponding flux.

---


### getIntervals

```
public java.util.Map<java.lang.String,Interval> getIntervals(java.util.Collection<java.lang.String> reactionNames)
```

:   Computes and returns the flux intervals of the reactions with the
    provided names.

    :   **Parameters:**: `reactionNames` - a collection of reaction names. **Returns:**: reaction names mapped to the computed interval of their corresponding flux.

---


### setFluxConstraint

```
public void setFluxConstraint(java.lang.String reactionName,
                              Interval c)
```

:   Changes the flux constraint of the reaction with the provided name.

    :   **Specified by:**: `setFluxConstraint` in interface `FluxCvComputer` **Overrides:**: `setFluxConstraint` in class `AbstractFluxIntervalComputer`
    :   **Parameters:**: `reactionName` - the name of the reaction whose flux constraint is to be changed.: `c` - the new constraint.

---


### setFluxConstraints

```
public final void setFluxConstraints(java.util.Map<java.lang.String,Interval> fluxConstraints)
```

:   Changes the flux constraints on more than one reaction.

    :   **Specified by:**: `setFluxConstraints` in interface `FluxCvComputer` **Overrides:**: `setFluxConstraints` in class `AbstractFluxIntervalComputer`
    :   **Parameters:**: `fluxConstraints` - reaction names mapped to their respective flux constraints.

---


### setRxnExprConstraint

```
public final void setRxnExprConstraint(MathExpr<java.lang.String> e,
                                       Interval c)
```

:   Changes the constraint on the provided mathematical expression
    of fluxes.

    :   **Specified by:**: `setRxnExprConstraint` in interface `FluxCvComputer` **Overrides:**: `setRxnExprConstraint` in class `AbstractFluxIntervalComputer`
    :   **Parameters:**: `e` - a matheamtical expression of reaction names.: `c` - the new constraint.

---


### setRxnExprConstraints

```
public final void setRxnExprConstraints(java.util.Map<? extends MathExpr<java.lang.String>,Interval> constraints)
```

:   Changes the constraints on more than one mathematical expression
    of fluxes.

    :   **Specified by:**: `setRxnExprConstraints` in interface `FluxCvComputer` **Overrides:**: `setRxnExprConstraints` in class `AbstractFluxIntervalComputer`
    :   **Parameters:**: `constraints` - mathematical expressions of reaction names mapped to their new respective constraints.

---


### restoreFluxConstraint

```
public final void restoreFluxConstraint(java.lang.String reactionName)
```

:   Restores the flux constraint for the reaction with the provided
    name to the value contained in the fluxome.

    :   **Specified by:**: `restoreFluxConstraint` in interface `FluxCvComputer` **Overrides:**: `restoreFluxConstraint` in class `AbstractFluxIntervalComputer`
    :   **Parameters:**: `reactionName` - the name of the reaction whose flux constraint is to be restored.

---


### restoreFluxConstraints

```
public final void restoreFluxConstraints(java.util.Collection<java.lang.String> reactionNames)
```

:   Restores the flux constraints for the provided reaction names to the
    values contained in the fluxome.

    :   **Specified by:**: `restoreFluxConstraints` in interface `FluxCvComputer` **Overrides:**: `restoreFluxConstraints` in class `AbstractFluxIntervalComputer`
    :   **Parameters:**: `reactionNames` - the reaction names whose flux constraints are to be restored.

---


### restoreFluxConstraints

```
public void restoreFluxConstraints()
```

:   Restores the flux constraints for all reactions in the fluxome to those
    values contained in the fluxome.

    :   **Specified by:**: `restoreFluxConstraints` in interface `FluxCvComputer` **Overrides:**: `restoreFluxConstraints` in class `AbstractFluxIntervalComputer`

---


### restoreRxnExprConstraint

```
public final void restoreRxnExprConstraint(MathExpr<java.lang.String> rxnNameExpr)
```

:   Restores the constraint on the provided flux expression to the value
    contained in the fluxome.

    :   **Specified by:**: `restoreRxnExprConstraint` in interface `FluxCvComputer` **Overrides:**: `restoreRxnExprConstraint` in class `AbstractFluxIntervalComputer`
    :   **Parameters:**: `rxnNameExpr` - the expression of reaction names whose contraint is to be restored.

---


### restoreRxnExprConstraints

```
public final void restoreRxnExprConstraints(java.util.Collection<? extends MathExpr<java.lang.String>> rxnNameExprs)
```

:   Restores the constraints on the provided expressions to the values
    contained in the fluxome.

    :   **Specified by:**: `restoreRxnExprConstraints` in interface `FluxCvComputer` **Overrides:**: `restoreRxnExprConstraints` in class `AbstractFluxIntervalComputer`
    :   **Parameters:**: `rxnNameExprs` - the reaction name expressions whose contraints are to be restored.

---


### restoreRxnExprConstraints

```
public final void restoreRxnExprConstraints()
```

:   Restores the constraints on all flux expressions to the values
    contained in the fluxome.

    :   **Specified by:**: `restoreRxnExprConstraints` in interface `FluxCvComputer` **Overrides:**: `restoreRxnExprConstraints` in class `AbstractFluxIntervalComputer`

---


### getRxnExprConstraints

```
public java.util.Map<MathExpr<java.lang.String>,Interval> getRxnExprConstraints()
```

:   Returns all current flux expression constraints.

    :   **Specified by:**: `getRxnExprConstraints` in interface `FluxCvComputer` **Overrides:**: `getRxnExprConstraints` in class `AbstractFluxIntervalComputer`
    :   **Returns:**: mathematical expressions of reaction names mapped to their respective constraint.

---


### getFluxConstraints

```
public java.util.Map<java.lang.String,Interval> getFluxConstraints()
```

:   Returns all current flux constraints.

    :   **Specified by:**: `getFluxConstraints` in interface `FluxCvComputer` **Overrides:**: `getFluxConstraints` in class `AbstractFluxIntervalComputer`
    :   **Returns:**: reaction names mapped to their respective constraint.

---


### getRxnExprConstraint

```
public Interval getRxnExprConstraint(MathExpr<java.lang.String> rxnNameExp)
```

:   Returns the current constraint on the provided expression.

    :   **Specified by:**: `getRxnExprConstraint` in interface `FluxCvComputer` **Overrides:**: `getRxnExprConstraint` in class `AbstractFluxIntervalComputer`
    :   **Parameters:**: `rxnNameExp` - the mathematical expression of reaction names whose constraint is to be returned. **Returns:**: the current constraint on the provided expression.

---


### getFluxConstraint

```
public Interval getFluxConstraint(java.lang.String rxnName)
```

:   Returns the current flux constraint on the reaction with the provided
    name.

    :   **Specified by:**: `getFluxConstraint` in interface `FluxCvComputer` **Overrides:**: `getFluxConstraint` in class `AbstractFluxIntervalComputer`
    :   **Parameters:**: `rxnName` - the name of the reaction whose flux constraint is to be returned. **Returns:**: the current flux constraint on the reaction with the provided name.


---


|  |  |  |
| --- | --- | --- |
| **Overview** \| **Package** \| **Class** \| **Use** \| **Tree** \| **Deprecated** \| **Index** \| **Help** | |  |
| **PREV CLASS**   **NEXT CLASS** | **FRAMES**    **NO FRAMES**     **All Classes** |  |
| SUMMARY: NESTED \| FIELD \| CONSTR \| METHOD | DETAIL: FIELD \| CONSTR \| METHOD |  |


---
